# White-Matter Lesions and Cortical Cerebral Blood Flow Evaluation by 3D Arterial Spin-Labeled Perfusion MRI in Asymptomatic Divers: Correlation with Patent Foramen Ovale Ocurrence

**DOI:** 10.3390/jcm12082866

**Published:** 2023-04-14

**Authors:** José Ángel Cabrera, Javier Urmeneta Ulloa, Mar Jímenez de la Peña, Margarita Rubio Alonso, Miguel López Gavilán, Silvia Bayona Horta, Gonzalo Pizarro, Karlos Simon, Teresa Migoya, Vicente Martínez de Vega

**Affiliations:** 1 Cardiology Department, Hospital Universitario Quirónsalud Madrid, 28223 Madrid, Spain; 2 Radiology Department, Hospital Universitario Quirónsalud Madrid, 28223 Madrid, Spain; 3 Faculty of Biomedical and Health Sciences, Universidad Europea de Madrid, 28670 Madrid, Spain; 4 Medical Imaging Unit, Rey Juan Carlos University, 28943 Madrid, Spain; 5 Cardiology Department, Hospital Ruber Juan Bravo, Grupo Quirónsalud, 28006 Madrid, Spain; 6 Diving Hispania Mazarrón, 30860 Murcia, Spain

**Keywords:** patent foramen ovale, white-matter lesions, divers, 3D arterial spin-labeled

## Abstract

Cerebral white-matter lesions (cWML) can be caused by dilation of Virchow–Robin spaces or may correspond to true lacunar ischemic lesions. The aim of our study was to evaluate in asymptomatic divers the relationship between the presence of patent foramen ovale (PFO) and cWML, as well as their possible effects on cortical cerebral blood flow (CBF) by magnetic resonance (MRI) through the arterial spin labeling (ASL) sequence. Transthoracic echocardiography was performed for the identification of PFO, and cerebral magnetic resonance including the 3D-ASL sequence for CBF quantification. Thirty-eight divers, with a mean age 45.8 ± 8.6 years, were included. Nineteen healthy volunteers, mean age 41 ± 15.2 years, served as the control group. A total of 28.9% of divers had completed more than 1000 dives. It was found that 26.3% of divers presented with PFO in the echocardiographic study. cWML was evidenced in 10.5% of diver MRI studies. There was no statistically significant relationship between the presence of PFO and cWML (*p* = 0.95). We observed a lower blood flow in all brain areas assessed by the 3D-ASL sequence in the group of divers, compared with the control group. We did not find statistical differences in CBF as a function of the presence or absence of PFO, number of dives, or cWML evidence.

## 1. Introduction and Background

Patent foramen ovale (PFO) is estimated to be present in approximately one quarter of the diving population [1] and has been associated with the development of cryptogenic stroke, platipnea ortodesoxia syndrome, and decompression sickness in divers and astronauts [2]. There is controversy about how to approach patients who present PFO and wish to continue diving, especially if they are asymptomatic and have not previously experienced decompression sickness [3,4,5,6].

Cerebral white-matter lesions (cWML) are commonly found in MR studies. cWML appears to increase with age although both the typical timing and clinical significance of their appearance in healthy persons remains unclear [7]. The prevalence varies according to the literature reviewed, ranging from 5.3% in a population of healthy subjects aged 16–65 [7], to 11–21% in adults aged around 64 and 94% at age 82 [8,9]. These lesions can be caused by dilation of Virchow–Robin spaces or may correspond to true lacunar ischemic lesions, requiring differentiation through the acquisition of a complete protocol that includes enhanced sequences in the T1 and FLAIR sequence [6]. cWMLs have been associated with an increased development of dementia, including Alzheimer’s disease [10], cognitive impairment, and impairment of motor and functional development in advanced age [11,12]. Their physiopathology is not fully established and small vessel ischemic changes secondary to arteriolosclerosis are accepted as the main etiology [13].

Flow quantification using 3D-ASL cerebral magnetic resonance imaging (3D-ASL MRI) is a recent technique that enables a non-invasive, quantitative assessment of cerebral cortical blood flow; in other words, of the grey matter [14]. The ASL sequence uses intra-arterial water as an endogenous contrast agent to visualize the perfusion of tissues and mathematical models to quantify cortical brain flow. Advantages over other flow quantification techniques, include a lack of invasiveness, as it does not require the use of intravenous contrast agents or radioactive agents. The information can be acquired during the same structural data acquisition session. ASL is especially useful for selected pathologies in which ASL is particularly sensitive, such as migraine, neurodegenerative disorders, or multiple sclerosis in which the hemodynamic disorders are initially subtle [15,16].

ASL is an MRI-sequence in continuous development, with multiple technical advances in the last years [17,18,19,20]. The International Society for Magnetic Resonance in Medicine (ISMRM) has created a guidance protocol [14,21] for specific clinical indications, as well as the procedure and postprocessing of these sequences, to increase its accuracy. One of these technical advances, is the multidelay p-ASL sequences used in our study, that allow for the acquisition of the most reliable CBF in a short period of time, which makes it compatible with daily clinical practice studies [22]. Unlike classic 2D sequences, it overcomes previous limitations such as low signal strength, limited spatial resolution, and rapid decay of spine labeling. It is, therefore, a highly attractive novel technique to objectively assess brain flow in different neurological pathologies, as well as for its subsequent follow-up.

Our goal is to assess the relationship between the presence of PFO and cWML in recreational and professional divers, as well as their possible effects on cerebral blood flow (CBF) quantified by a perfusion study with MRI using the ASL sequence.

## 2. Materials and Methods

### 2.1. Study Population

We included 38 consecutive asymptomatic recreational and professional divers studied at our center. Nineteen healthy volunteers with no significant differences in age and no known cardiovascular risk factors were taken as the control group.

Among the inclusion criteria were: age between 18 and 60 years old, divers with at least 100 dives at depths greater than 20 m, and/or professional divers, without prior cryptogenic stroke, platipnea ortodesoxia syndrome, or decompression sickness, and with less than two classic cardiovascular risk factors for atherosclerosis. Exclusion criteria included: patients with known cerebrovascular or cardiovascular disease, pregnancy or lactation, and severe claustrophobia, as well as the usual exclusion criteria for conventional MRI: carriers of cardiac electronic devices (pacemakers and defibrillators), cochlear implants, non-MR compatible vascular clips, non-MR compatible cardiac valve prostheses, extensive tattoos, ocular metallic foreign bodies, and other high-risk areas (intracranial, spinal canal, large vessels, liver).

Participants were divided into four groups according to the total number of dives performed: group 1 (less than 200 dives), group 2 (between 200 and 500 dives), group 3 (between 500 and 1000 dives), and group 4 (more than 1000 dives). The type of diving practiced was taken into consideration, with dives being divided into two categories: recreational and professional.

Transthoracic echocardiography was performed using Vivid 9 ultrasound (General Electric Healthcare, Milwaukee, USA) with agitated saline to identify patent foramen ovale by passing bubbles into left cavities during the first three heartbeats [2]. These were classified into three groups according to the number of bubbles detected: slight passage (less than 10 bubbles), moderate passage (between 10 and 20 bubbles), and severe passage (more than 20 bubbles) [2]. Finally, a brain MRI was performed in all participants to detect the cWML.

Before performing the tests, they were explained in detail to each of the patients, who were required to sign a common informed consent form. The study protocol was compliant with the Declaration of Helsinki and received approval from the local medical ethics committee.

### 2.2. Cerebral Magnetic Resonance

**Figure 1:**
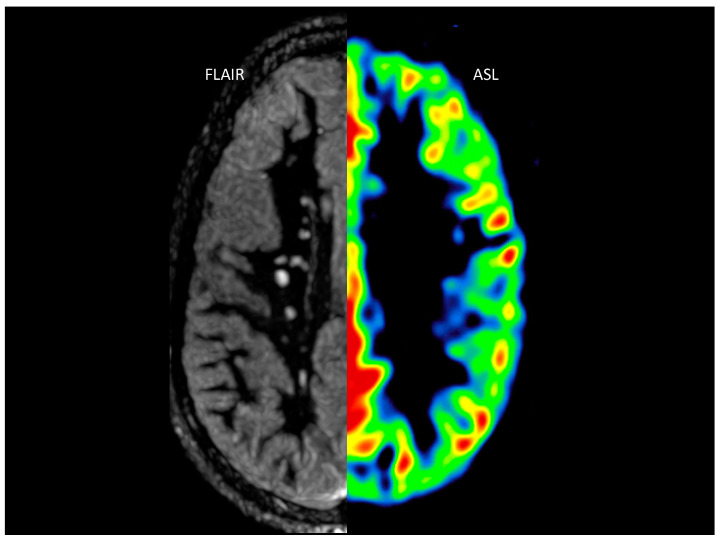
Combination of FLAIR sequence (**left**) for assessment of white-matter lesions and ASL perfusion sequence (**right**) for quantification of cerebral cortical flow.

Brain MRI studies were performed on an advanced 1.5T MR450 (General Electric Healthcare, Milwaukee), using an 8-channel phased array head antenna. Acquisition included a structural protocol with T1 3D-SPGR isovolumetric sequences (TE: 1 ms; TR: automatic; flip angle 10, 31.25 Hz, FOV: 28, cut thickness: 1.4 mm; phase FOV: 0.8; 2NEX; matrix: 228 × 228) and 3D FLAIR-CUBE (Figure 1) (TE: 124 ms; TR: 6000; TI: 1866; bandwidth: 31.25 Hz, FOV: 24, cut thickness: 1.2 mm; phase FOV: 0.9; 1NEX; matrix: 256 × 256).

A prototype non-contrast perfusion sequence called pseudo-continuous arterial spin labeling (p-ASL) [14] was added in the control and study subjects (Figure 1) based on the spin labeling technique using a 3D acquisition sequence, designed to improve the signal/noise ratio, reduce artifacts, and acquire multiple perfusion and post-labeling time maps, with the following parameters: TE: 11.5 ms; TR: 5981 ms, post-labeling delay: 1000 ms, perfusion labeling time: 3500 ms, number of delays: 7 (1000 ms, 1220 ms, 1480 ms, 1780 ms, 2150 ms, 2630 ms, and 3320 ms), bandwidth: 62.5 kHz; flip angle: 155, FOV: 22 cm; spiral acquisition: 640 points × 4 arms, reconstructed image: 128 × 128, slice thickness: 4.5 mm).

## 3. Data Analysis

MR studies were analyzed by an expert neuroradiologist, blind to the clinical and echocardiographic data. The number of white-matter lesions in each patient was quantified, and the study participants were divided into three categories (no lesions, ischemic lesions, and dilated Virchow–Robin spaces) in the 3D-FLAIR CUBE sequences.

To evaluate cerebral cortical flow in these participants, areas of interest were the superior, middle, inferior, and internal frontal gyrus. These areas are related to disability and cognitive dysfunction in working memory and processing speed, alterations found in professionals with many years of diving experience [23]. The caudate nucleus and the thalamus were also evaluated, as basal ganglia are involved in all brain regulation processes. Data obtained from the perfusion maps were the transit-time-corrected cerebral blood flow (CBF) and the bolus arrival time (BAT), also called arterial transit delay time, defined as the time from the labeling of blood in the feeding arteries to its first arrival in the capillary network of the voxel of interest. From the 3D-T1 weighted sequences, we computed cerebral volume (CV), a basic parameter in the measurement of neurodegenerative pathology.

### Postprocessing

**Figure 2:**
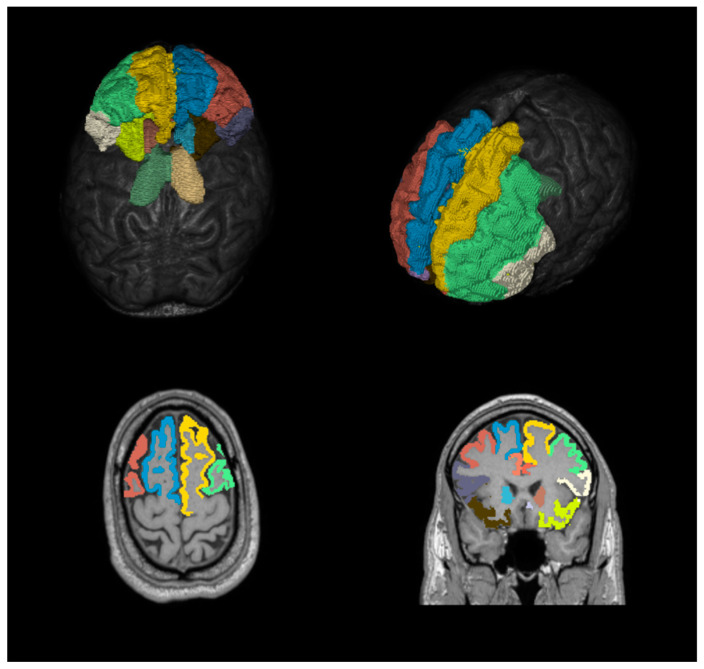
Volumetric reconstruction (**upper**) and segmentation of 3D images (**lower**) enhanced in T1, color map showing the cortical areas and basal ganglia evaluated.

Brain parcellation. 3D-T1 images were parcellated using FreeSurfer (https://surfer.nmr.mgh.harvard.edu/) (accessed on 11 November 2019). The result of this automatic parcellation is a label map containing a large number of brain regions, along with specific anatomical information—volume, area, and cortical thickness—for each segmented region (Figure 2). The label map produced by FreeSurfer has different dimensions than the original T1 image and was later co-registered to the T1 image to recover the original resolution. Among all the brain regions present in the output label map, 18 regions of interest (ROI) considered significant to our study were selected.

**Figure 3:**
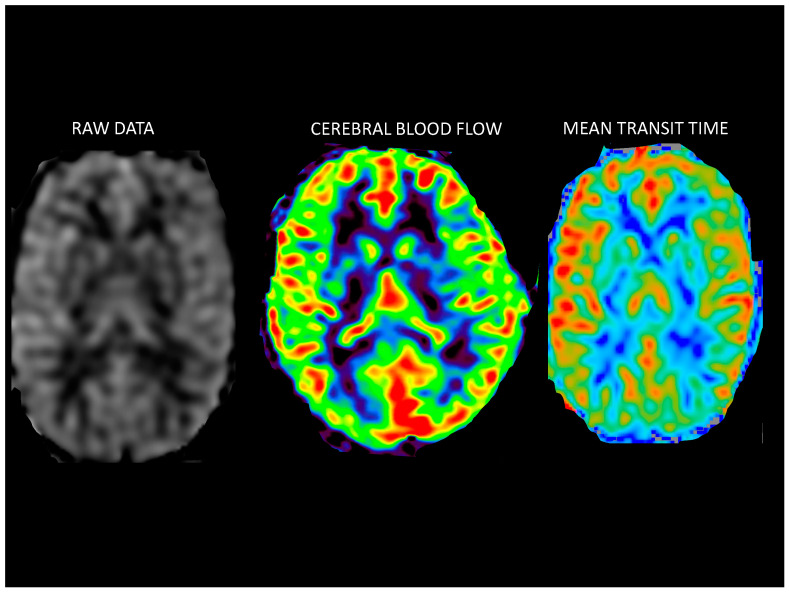
ASL perfusion sequence processing.

Perfusion and BAT quantification. The ASL image was co-registered to the T1 image and the resulting transformation was applied to both CBF and BAT volumes. Mean perfusion (mL/100 g/min) and bolus arrival time (ms) were then quantified from the registered CBF and BAT volumes for each ROI of interest (Figure 3).

Statistical analysis was performed using SPSS version 25 software (IBM, New York, NY, USA). Categorical variables are presented as numbers and percentages, and continuous variables as mean ± standard deviation, after checking the fit to a normal distribution using the Shapiro–Wilk test. For comparisons between categorical variables, Pearson’s χ^2^ test or Fisher’s exact test was used when necessary. For comparisons of quantitative variables, Student’s t was used for independent samples after checking the homogeneity of the variance. *p* values of less than 0.05 were considered statistically significant.

## 4. Results

### 4.1. Population

A total of 38 asymptomatic divers with a mean age of 45.8 ± 8.6 years were included. The control group was made up of 19 healthy volunteers with a mean age of 41 ± 15.2 years. There were no significant differences in the age of the two groups (*p* = 0.21). In the divers group, there were significantly more males (32; 84.2%) than in the control group (12; 63.2%) (*p* < 0.001).

Regarding cardiovascular risk factors, healthy volunteers were free of any pathology. On the other hand, in the group of divers, the most frequent risk factor was positive smoking habit in 21.1% [8], followed by arterial hypertension in 15.8% [6], and, finally, dyslipidemia in 7.9% [3] of participants (Table 1).

In terms of the number of dives, all divers had made at least 100 dives, to depths greater than 20 m, and approximately one quarter of the population of divers, 11 divers (28.9%), had completed more than 1000 dives. A total of 16 divers (42.1%) were professionals (Table 1).

### 4.2. Patent Foramen Ovale and White-Matter Brain Lesions

Transthoracic echocardiography detected patent foramen ovale in ten of the divers (26.3%), and in eight of these (80%), the bubble passage was slight (<10). In relation to the other echocardiographic findings, left/right ventricular function was preserved without repercussions for ventricular cavities, and no significant valvular diseases were observed.

With cerebral MRI, no lesions were observed in the control group; four divers (10.5%) presented with cWML and twelve (31.6%) with dilated Virchow–Robin spaces (with no pathological significance) (Table 1). In our sample, there was no statistically significant relationship between the presence of PFO and cWML (*p* = 1).

### 4.3. Measurement of Cerebral Blood Flow Using 3D-pASL

**Figure 4:**
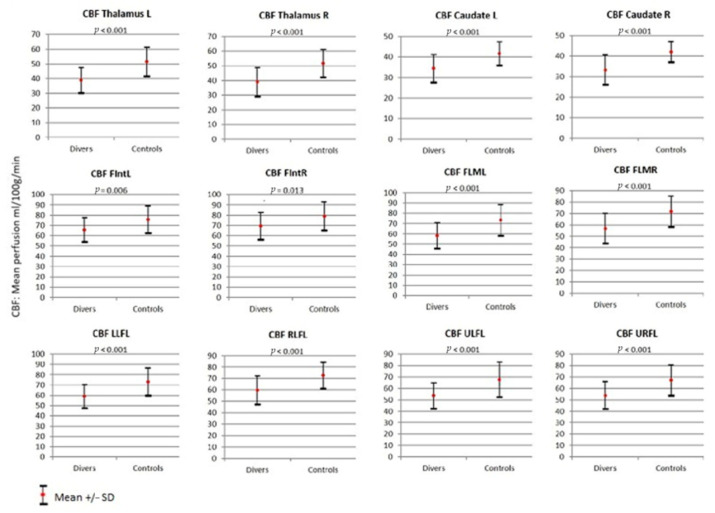
Determination of cortical cerebral blood flow measured through the 3D-pASL sequence. Comparison of findings in the divers group versus control group. Abbreviations: CBF: cerebral blood flow; L: left; R: right; FIntL: frontal internal left; FIntR: frontal internal right; FLML: frontal lobe, middle left; FLMR: frontal lobe, middle right; LLFL: left lower frontal lobe; RLFL: right lower frontal lobe; ULFL: upper left frontal lobe; URFL: upper right frontal lobe.

With regards to the determination of cortical cerebral blood flow measured through the 3D-pASL sequence, in the group of divers, there was less blood flow in all cerebral areas evaluated compared with the control group. Differences in CBF between the groups were statistically significant in all areas studied (Table 2, Figure 4). With respect to transit time, we only found statistically significant differences at the level of the medial inferior frontal lobe (MIFL), with a greater delay in cerebral blood flow compared to controls (1454.7 vs. 1383.8 msg *p* = 0.032) (Table 2).

On the other hand, when analyzing cerebral blood flow in the group of divers according to presence or absence of PFO, we did not find statistically significant differences in the evaluated cerebral areas (Table S1). We also analyzed the relationship between age and cerebral perfusion and found a lower cerebral blood flow and longer cerebral transit time in older individuals (Table S2). This relationship was observed in most of the cerebral regions examined by the 3D-pASL sequences.

When comparing divers with more than 1000 dives with those with less than 1000 dives, we did not observe any statistically significant differences in CBF quantification (Table S3), nor did we find any differences in CBF between those who presented cWML and those who did not.

## 5. Discussion

It is suspected that those who present with a transient or permanent right–left shunt such as PFO during the Valsalva maneuver, are susceptible to decompression sickness and clinical manifestations secondary to paradoxical embolism, ranging from musculoskeletal symptoms to severe neurological pathology [24].

The causal relationship between the existence of PFO in asymptomatic divers and the presence of cWML is a subject of ongoing debate. Initially, some groups identified a greater number of ischemic lesions in divers [25,26], including a positive relationship between the existence of PFO and the presence of cWML [26]. However, the latest studies carried out with a complete acquisition protocol of cerebral MRI including T1–T2 assessment and FLAIR sequences, show negative correlation results between presenting with PFO and evidence of cWML [5,6,27,28]. This highlights the need to differentiate these from dilated Virchow–Robin spaces, visualized only in T2 sequences, and considered as a non-pathological finding, as the positive relationship reported in the initial studies can probably be attributed to the joint assessment of these two findings [6].

In our sample, we found a higher number of cWML in divers than in healthy volunteers (10.5 vs. 0%, *p* = 0.04). At the same time, we witnessed a greater number of dilated Virchow–Robin spaces (31.6% vs. 0%, *p* = 0.04), until now considered a non-pathological finding. However, according to the latest studies, we did not find a significant relationship between the presence of PFO and having cWML in the group of divers (*p* = 0.950), nor was it related to hypertension, dyslipidemia, or positive smoking habit. Therefore, according to our results, the presence of cWML is more frequent in divers, although this relationship is not explained by the existence of PFO in this population. On comparing the composition of the groups, the only statistically significant difference was related to sex, with more males in the group of divers (84.2 vs. 36.8%, *p* < 0.01). Regarding the age factor, there were no significant differences between the group of divers and the control group, so the findings cannot be explained by the age of the study population.

Genuine cWML are characterized by demyelination, axonal loss, and rarefaction, mostly attributed to ischemia and associated with arteriolosclerosis [13]. The presence of cWML has been related to a greater development of Alzheimer-type dementia and cognitive deterioration in healthy people over 60 years of age [29,30]. This lesional white-matter load may influence cortical cerebral blood flow as previously reported [31,32,33,34]. It can, therefore, be inferred that the presence of cWML can also have a deleterious effect on the evolution of the cognitive state of divers, although this hypothesis has yet to be confirmed.

The measurement of CBF in the grey matter can be evaluated quantitatively with ASL sequences, which have demonstrated an excellent reproducibility in healthy patients [22,35], and even more so with the multidelay p-ASL sequence [36]. Its application is interesting in subjects with cWML, to determine whether the cortical CBF related to these lesions is significantly reduced compared to normal tissue [22,32].

To our knowledge, this study demonstrates for the first time reduced values of cerebral blood flow in all brain regions of participating divers evaluated by 3D-ASL sequences, compared to healthy volunteers. This finding is highly significant due to the large number of individuals worldwide who practice this sport. Although we did not find differences in cerebral blood flow in divers with more than 1000 dives compared with those with a smaller total number, we did find a trend towards a statistical significance of presenting cWML in the participants with the most diving experience (Table S4). Cerebral cortical blood flow was found to be reduced in all divers equally regardless of the number of dives, the number of cWML, and the evidence of PFO. Therefore, according to our results, it is probably more the practice of the sport itself, regardless of evidence of PFO or dive frequency, that determines a lower cortical brain flow in this population.

This reduction in flow appears to be independent of that occurring in certain areas with age [37], or of concomitant factors such as diabetes [38] or high blood pressure [39]. In our study, the mean age of divers was only 45.8 ± 8.6 years (no statistically significant differences to the control group), so age does not seem to have influenced our results. On the other hand, we did not have a single diabetic patient, and for arterial hypertension, we did not obtain a statistically positive correlation between presenting cWML and hypertension.

In the literature, cWML could be the result of prolonged hyperbaric exposure, representing repetitive ischemic insults in the brain microvasculature. The combination with a short, but repeated period of more generalized white-matter metabolic stress or demand might explain these cWML, especially in distal, watershed, or peripheral areas. These hemodynamic changes are different in each patient, although over time they would impair the white and gray-matter perfusion [40,41,42,43,44].

A high cerebral metabolic demand under normal conditions requires a blood flow of approximately 45–50 mL 100 g^−1^ min^−1^, ranging from 20 mL 100g^−1^ min^−1^ in the white matter, to 70 mL 100 g^−1^ in the grey matter [45]. Brain self-regulation is a neuroprotective mechanism that helps to maintain cerebral blood flow when there are important hemodynamic changes that require immediate compensation. Among the factors that can alter cerebral blood flow are: blood pressure, cerebral perfusion pressure, temperature, partial pressure of carbon dioxide (PaCO_2_), partial pressure of arterial oxygen (PaO_2_), blood viscosity, and sympathetic and parasympathetic activity of the nervous system in general [46]. Considering the above, a possible hypothesis to explain our findings of decreased cortical cerebral blood flow in divers would be an impairment or slowdown of brain hemodynamic self-regulation, or a negative compensatory self-regulation, in response to repeated hyperbaric conditions during submergence.

Finally, it is important to point out that we did not find a statistically significant difference in the quantification of CBF in divers with cWML compared with those without cWML, or in those participants who presented with Virchow–Robin spaces. However, as previously mentioned, decreased brain flow has been associated with greater cognitive impairment in older age [29,30], and a possible link between long-term cognitive impairment and the presence of a reduced brain flow in this population is of concern. Therefore, the possible implications of a reduction in CBF found in divers must be evaluated with caution. Subsequent studies are required to evaluate the long-term clinical repercussions of presenting with a lower CBF in this scenario, and thereby generate conclusions as well as recommendations for such a widely practiced activity.

## 6. Limitations

There are several limitations to our study. In the first place, we did not perform imaging follow-up. Furthermore, patent foramen ovale was only assessed by transthoracic echocardiography with agitated saline, justified by the refusal of patients to undergo a transesophageal echocardiography (a semi-invasive study, with greater risks and discomfort for the group studied), and taking into consideration that TTE with contrast saline is an excellent alternative. At the same time, the sample of divers with PFO in our population mostly had a slight passage of bubbles (80% with <10 bubbles), so the results should be taken with caution, without being able to extrapolate our results to divers with significant shunting. Finally, although this is the first study to assess cerebral blood flow using 3D-PASL in asymptomatic divers without previous decompression syndrome, the number of participants was relatively small.

## 7. Conclusions

We showed a lower cortical cerebral blood flow by the MRI 3D-pASL sequence in all evaluated brain areas of asymptomatic divers. There was a greater presence of cWML in divers unrelated to the existence of PFO in this population.

## Figures and Tables

**Table 1:** Baseline characteristics.

	Divers	Control Group
Age (years)	45.8 ± 8.6	41 ± 15.2
Male	32/38 (84.2%)	12/38 (63.2%)
Cardiovascular RF:		0/19 (0%)
-Hypertension	6/38 (15.8%)	
-Dyslipidemia	3/38 (7.9%)	
-Smoking	8/38 (21.1%)	
Echocardiography:		N/A
-Patent foramen ovale	10/38 (26.3%)	
-Degree of PFO		
<10 bubbles	8/10 (80%)	
10–20 bubbles	1/10 (10%)	
>20 bubbles	1/10 (10%)	
Number of dives		N/A
<200	11/38 (28.9%)	
200–500	12/38 (31.6%)	
500–1000	4/38 (10.5%)	
>1000	11/38 (28.9%)	
Type of diving		N/A
-Recreational	22/38 (57.9%)	
-Professional	16/38 (42.1%)	
MRI:		
-Ischemic defects	4/38 (10.5%)	0/38 (0%)
-Virchow–Robin	12/38 (31.6%)	0/38 (0%)
-No findings	22/38 (57.9%)	38/38 (100%)

Abbreviations: RF: risk factors; PFO: patent foramen ovale; CMR: cerebral magnetic resonance.

**Table 2:** Brain blood flow and transit time. Differences between divers and controls.

	Divers (n = 38) Mean ± SD	Controls (n = 19) Mean ± SD	*p*
Age	45.84 ± 8.58	41.05 ± 15.24	0.215
CBF THALAMUS L	38.91 ± 10.74	51.57 ± 9.79	<0.001
CBF THALAMUS R	38.97 ± 9.69	51.57 ± 9.54	<0.001
CBF CAUDATE L	34.35 ± 6.72	41.58 ± 5.8	<0.001
CBF CAUDATE R	33.25 ± 7.18	42.15 ± 4.98	<0.001
CBF FIntL	65.95 ± 11.62	75.79 ± 13.33	0.006
CBF FIntR	69.44 ± 13.06	79.05 ± 13.65	0.013
CBF FLML	58.25 ± 12.51	73.38 ± 15.21	<0.001
CBF FLMR	56.88 ± 13.16	71.87 ± 13.58	<0.001
CBF LLFL	59.04 ± 11.63	73.24 ± 13.49	<0.001
CBF RLFL	59.83 ± 12.26	72.61 ± 11.41	<0.001
CBF ULFL	53.79 ± 11.29	67.72 ± 15.3	<0.001
CBF URFL	54.13 ± 11.9	67.31 ± 13.19	<0.001
TT URFL	1353.59 ± 128.17	1339.77 ± 89.76	0.676
TT ULFL	1344.63 ± 145.44	1340.79 ± 81.96	0.915
TT RLFL	1263.81 ± 146.61	1249.69 ± 104.41	0.71
TT LLFL	1251.58 ± 139.16	1241.44 ± 84.27	0.772
TT FLMR	1452.69 ± 169.67	1400.69 ± 75.76	0.116
TT FLML	1454.68 ± 168.61	1383.85 ± 73.58	0.032
TT FIntR	1198.93 ± 87.99	1191.55 ± 88.16	0.767
TTFIntL	1208.42 ± 82.60	1181.89 ± 72.04	0.467
TT CAUDATE R	1220.75 ± 91.03	1188.55 ± 82.3	0.2
TT CAUDATE L	1210.77 ± 102.33	1187.33 ± 93.77	0.406
TT THALAMUS R	1297.75 ± 124.02	1290.8 ± 106.67	0.836
TT THALAMUS L	1299.42 ± 124.84	1290.83 ± 101.66	0.796

Abbreviations: CBF: cerebral blood flow; L: left; R: right; FIntL: frontal internal left; FIntR: frontal internal right; FLML: frontal lobe, middle left; FLMR: frontal lobe, middle right; LLFL: left lower frontal lobe; RLFL: right lower frontal lobe; ULFL: upper left frontal lobe; URFL: upper right frontal lobe; TT: transit time.

## Data Availability

Data supporting the findings of this study can be found in the article or its Supplementary Material, and detailed data are available from the corresponding author upon reasonable request.

## Supplementary Materials

The following supporting information can be downloaded at: https://www.mdpi.com/article/10.3390/jcm12082866/s1, Table S1: Cerebral blood flow correlation according to PFO in the group of divers; Table S2: Cerebral blood flow correlation according to age; Table S3. Differences in CBF quantification between divers with ≥1000 dives (n = 11) and divers with <1000 dives (n = 27); Table S4: Presence of cWML in divers with <1000 dives and divers with ≥1000 dives.

## Abbreviations

A4C	Apical 4-Chamber
BAT	Bolus arrival time
CBF	Cerebral blood flow
CMR	Cerebral magnetic resonance
CRF	Cardiovascular risk factors
CV	Cerebral volume
CWML	Cerebral white-matter lesions
FL	Frontal lobe
FLAIR	Fluid-attenuated inversion recovery
FOV	Field of view
FSE	Fast spin echo
PFO	Patent foramen ovale
ROI	Regions of interest
SCUBA	Self-contained underwater breathing apparatus
TE	Time echo
TR	Time repetition
UBOs	Unidentified bright brain objects
3D-ASL	Three-dimensional arterial spin labeling
3D-SPGR	Three-dimensional spoiled gradient recalled acquisition in steady state
