# Supplementary material for: White-Matter Lesions and Cortical Cerebral Blood Flow Evaluation by 3D Arterial Spin-Labeled Perfusion MRI in Asymptomatic Divers: Correlation with Patent Foramen Ovale Ocurrence
Source: J Clin Med. 2023 Apr 14;12(8):2866. doi: 10.3390/jcm12082866 (PMC10141148; doi:10.3390/jcm12082866)
Supplement: Supplementary file 1 [file jcm-12-02866-s001.zip › jcm-2267431-supplementary.pdf]

Journal of
Clinical Medicine

MDPI

Supplementary Materials

Table S1. Cerebral blood flow correlation according to PFO in the group of divers.

	PFO		*p*
	**Yes (n=10)**	**No (n=28)**	
	**mean±SD**	**mean±SD**	
CBF THALAMUS L	34.94±8.96	40.33±11.11	0.18
CBF THALAMUS R	34.18±8.48	40.68±9.66	0.07
CBF CAUDATE L	32.06±5.08	35.17±7.12	0.21
CBF CAUDATE R	31.07±6.23	34.03±7.44	0.27
CBF FIntL	65.20±10.77	66.22±12.08	0.82
CBF FIntR	72.63±13.23	68.30±13.05	0.38
CBF FLML	61.49±11.08	57.10±12.97	0.35
CBF FLMR	59.94±12.22	55.78±13.52	0.40
CBF LLFL	60.61±10.37	58.49±12.18	0.63
CBF RLFL	61.17±11.31	59.35±12.75	0.69
CBF ULFL	53.61±8.80	53.86±12.20	0.95
CBF URFL	54.98±9.98	53.83±12.67	0.80
TT URFL	1360.63±80.29	1351.07±142.61	0.84
TT ULFL	1370.92±82.65	1335.24±162.37	0.51
TT RLFL	1286.45±106.49	1255.72±159.43	0.58
TT LLFL	1274.82±105.84	1243.28±150.10	0.55
TT FLMR	1479.72±141.87	1443.03±179.93	0.56
TT FLML	1494.40±148.50	1440.50±175.55	0.39
TT FIntR	1172.67±60.82	1208.31±95.03	0.28
TTFIntL	1196.71±45.63	1212.61±92.67	0.06
TT CAUDATE R	1220.27±67.62	1220.92±99.15	0.98
TT CAUDATE L	1194.29±69.26	1216.65±112.31	0.56
TT THALAMUS R	1300.72±118.81	1296.69±127.94	0.93
TT THALAMUS L	1316.69±110.01	1293.25±131.04	0.62

Abbreviations: CBF: Cerebral blood flow; L: left; R: right; FIntL: Frontal internal left; FIntR: Frontal internal right; FLML: Frontal lobe. middle left; FLMR: Frontal lobe. middle right; LLFL: Left lower frontal lobe; RLFL: Right lower frontal lobe; ULFL: Upper left frontal lobe; URFL: Upper right frontal lobe; TT: Transit time.

Table S2. Cerebral blood flow correlation according to age.

	**Divers (n=38)**		**Controls (n=19)**	
	Pearson's *r	***p***	***Pearson's *r***	***p***
CBF THALAMUS L	-0.29	0.07	0.08	0.73
CBF THALAMUS R	-0.28	0.08	0.08	0.74
CBF CAUDATE L	-0.36	0.03	-0.30	0.21
CBF CAUDATE R	-0.39	0.01	-0.18	0.63
CBF FIntL	-0.44	0.01	-0.53	0.02
CBF FIntR	-0.48	<0.001	-0.56	0.01
CBF FLML	-0.65	<0.001	-0.54	0.02
CBF FLMR	-0.53	<0.001	-0.57	0.01
CBF LLFL	-0.62	<0.001	-0.42	0.07
CBF RLFL	-0.47	<0.001	-0.39	0.10
CBF ULFL	-0.47	<0.001	-0.62	0.01
CBF URFL	-0.48	<0.001	-0.64	0.01
TT URFL	0.16	0.34	0.027	0.91
TT ULFL	0.13	0.42	-0.06	0.78
TT RLFL	0.10	0.54	0.160	0.52
TT LLFL	0.08	0.62	0.05	0.84
TT FLMR	0.12	0.48	0.14	0.55
TT FLML	0.09	0.60	0.17	0.47
TT FIntR	0.15	0.38	-0.28	0.25
TTFIntL	0.17	0.30	-0.41	0.08
TT CAUDATE R	0.24	0.15	-0.14	0.57
TT CAUDATE L	0.38	0.02	-0.11	0.66
TT THALAMUS R	0.18	0.29	0.22	0.37
TT THALAMUS L	0.22	0.18	0.21	0.40

Abbreviations: CBF: Cerebral blood flow; L: left; R: right; FIntL: Frontal internal left; FIntR: Frontal internal right; FLML: Frontal lobe. middle left; FLMR: Frontal lobe. middle right; LLFL: Left lower frontal lobe; RLFL: Right lower frontal lobe; ULFL: Upper left frontal lobe; URFL: Upper right frontal lobe; TT: Transit time. *Pearson´s correlation coefficient.

Table S3. Differences in CBF quantification between divers with ≥ 1000 dives (n=11) and divers with < 1000 dives (n=27).

	**<1000 dives mean±SD**	**≥1000 dives mean±SD**	***p***
CBF THALAMUS L	38.39±11.06	40.18±10.32	0.65
CBF THALAMUS R	38.90±10.03	39.12±9.28	0.95
CBF CAUDATE L	33.98±6.85	35.25±6.64	0.61
CBF CAUDATE R	32.97±7.61	33.93±6.30	0.72
CBF FIntL	67.55±12.38	62.02±8.77	0.19
CBF FIntR	70.05±13.90	67.94±11.19	0.66
CBF FLML	58.36±12.47	57.99±13.21	0.93
CBF FLMR	56.45±12.96	57.92±14.21	0.76
CBF LLFL	59.40±11.89	58.17±11.49	0.77
CBF RLFL	60.18±12.45	58.97±12.35	0.79
CBF ULFL	53.72±11.44	53.97±11.45	0.95
CBF URFL	54.27±12.38	53.79±11.22	0.91
TT URFL	1354.72±141.81	1350.81±92.12	0.93
TT ULFL	1349.66±163.79	1332.29±91.01	0.74
TT RLFL	1274.35±166.21	1237.94±81.62	0.49
TT LLFL	1264.25±158.07	1220.46±72.02	0.39
TT FLMR	1455.90±187.69	1444.81±121.78	0.86
TT FLML	1465.41±185.06	1428.35±122.80	0.55
TT FIntR	1204.41±100.25	1185.48±47.27	0.55
TTFIntL	1222.87±89.16	1172.98±51.29	0.53
TT CAUDATE R	1218.19±78.78	1227.05±120.24	0.79
TT CAUDATE L	1220.51±104.53	1186.86±97.21	0.37
TT THALAMUS R	1318.06±137.32	1247.89±63.49	0.11
TT THALAMUS L	1309.68±135.53	1274.24±94.47	0.44

Abbreviations: CBF: Cerebral blood flow; L: left; R: right; FIntL: Frontal internal left; FIntR: Frontal internal right; FLML: Frontal lobe. middle left; FLMR: Frontal lobe. middle right; LLFL: Left lower frontal lobe; RLFL: Right lower frontal lobe; ULFL: Upper left frontal lobe; URFL: Upper right frontal lobe; TT: Transit time. SD: Standard deviation.

Table S4. Presence of cWML in divers with <1000 dives and divers with ≥1000 dives.

		**<1000 dives (n=27)**	**≥1000 dives (n=11)**	***p***
cWML	Yes	1 (3.7%)	3 (27.3%)	0.065
	No	26 (96.3%)	8 (72.5%)	
